# Conformational Solvation Studies of LIGNOLs with Molecular Dynamics and Conductor-Like Screening Model

**DOI:** 10.3390/ijms13089845

**Published:** 2012-08-07

**Authors:** Thomas Sandberg, Patrik Eklund, Matti Hotokka

**Affiliations:** 1 Centre of Excellence for Functional Materials, Physical Chemistry, Åbo Akademi University, Porthansgatan 3–5, 20500 Åbo (Turku), Finland; E-Mail: mhotokka@abo.fi; 2 Organic Chemistry, Åbo Akademi University, Biskopsgatan 8, 20500 Åbo (Turku), Finland; E-Mail: paeklund@abo.fi

**Keywords:** conformation, chiral 1, 4-diol, lignan, LIGNOL, molecular dynamics, GROMACS, COSMO

## Abstract

Molecular dynamics (MD) simulations were performed on sterically hindered *α*-conidendrin-based chiral 1,4-diols (LIGNOLs) from the naturally occurring lignan hydroxymatairesinol (HMR) using the GROMACS software. The aim of this study was to explore the conformational behaviour of the LIGNOLs in aqueous solution adopting the TIP4P model. The topologies of the LIGNOLs were constructed manually and they were modeled with the OPLS-AA force field implemented in GROMACS. The four most relevant torsional angles in the LIGNOLs were properly analyzed during the simulations. The determining property for the conformation preferred in aqueous solution was found to be the lowest energy in gas phase. The solvation effects on the LIGNOLs were also studied by quantum chemical calculations applying the COnductor-like Screening MOdel (COSMO). The hydration studies of the MD simulations showed that several of these LIGNOLs, produced from a renewable source, have a great potential of acting as chiral catalysts.

## 1. Introduction

The forest industries are developing new innovative products in addition to the traditional bulk products. A promising raw material for added-value products consists of lignans that are extracted in chemical pulping from residual knots.

The anticarcinogenic and antioxidative lignan hydroxymatairesinol (HMR) is found in large amounts in the knots of Norway spruce (Picea abies) [1]. It has been used in the synthesis of TADDOL-like *α*-conidendrin-based chiral 1,4-diols (LIGNOLs) [2] with the same functionality as TADDOLs [3,4] or BINOLs [5], which are often used as ligands for transition metal catalysed asymmetric synthesis. They have hindered structures containing two adjacent stereocenters, resulting in a fixed angle between the metal-complexing hydroxyl groups.

HMR has recently been studied by quantum chemical calculations [6,7] and by molecular dynamics (MD) [8]. The structures of the LIGNOLs included in this study have been quantum chemically optimized [2], and in this study MD simulations are used to explore the conformational changes of the LIGNOLs in aqueous solution. Such an analysis has to the best of our knowledge not been performed before.

Solvation effects are important to include in quantum chemical calculations because solvents play a role in the structures of many molecules. This can be done by using implicit solvation models such as, e.g., the COnductorlike Screening MOdel (COSMO) [9,10]. In a previous study [8] on the structurally quite similar lignan HMR, solvent effects calculated by COSMO were compared to solvent effects by the Polarized Continuum Model (PCM) [11–13], and COSMO was found to give more credible relative energies than PCM.

## 2. Results and Discussion

**Figure 1:**
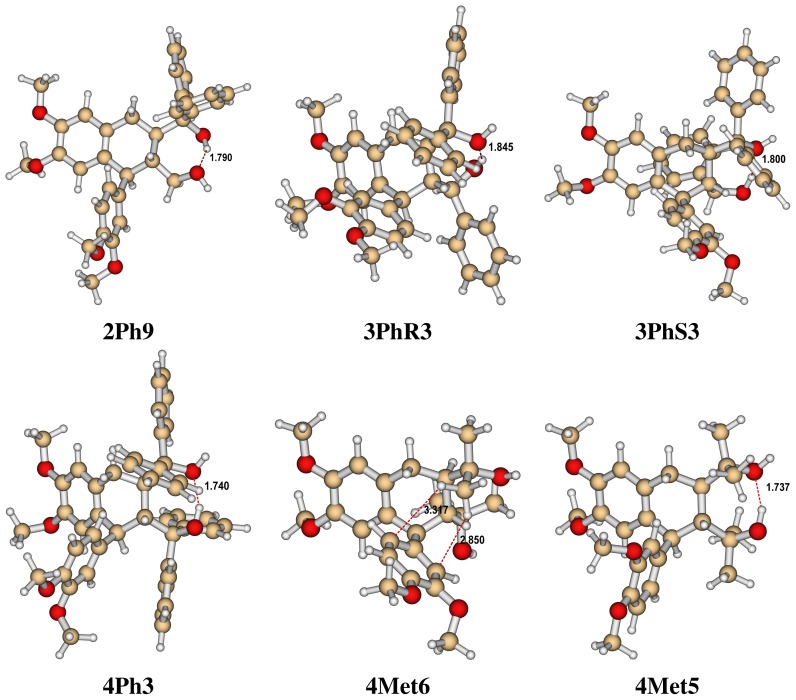
The minimum energy structure for each LIGNOL: Diphenyl (top left), triphenyl(R) (top middle), triphenyl(S) (top right), tetraphenyl (bottom left), tetramethyl (bottom middle) and tetramethyl that could work as catalyst (bottom right) [2].

In this study the following chiral 1,4-diols (LIGNOLs) have been investigated: 1,1-diphenyl (**2Ph**), two diastereomers of 1,1,4-triphenyl (**3PhR**, **3PhS**), 1,1,4,4-tetraphenyl (**4Ph**) and 1,1,4,4-tetramethyl (**4Met**) 1,4-diol. The minimum energy structure for each LIGNOL is shown in Figure 1 [2]. The code for the conformations is adopted from [2].

**Figure 2:**
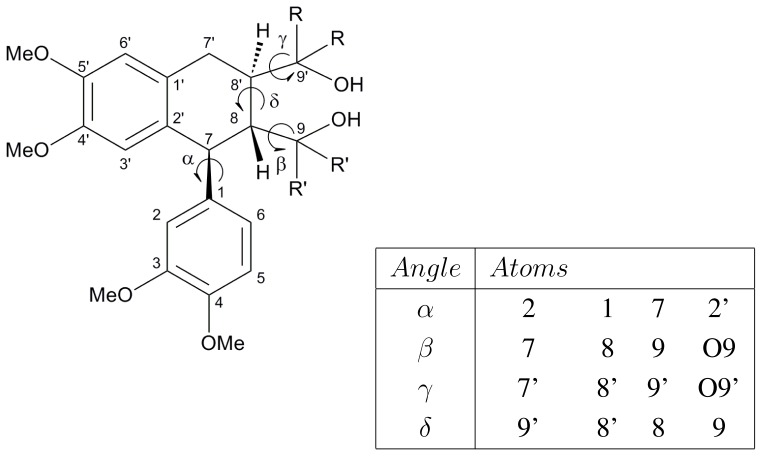
The four most relevant torsional angles and the numbering of atoms [2]. For tetramethyl 1,4-diol, R = R*′* = methyl. For the others, R = phenyl, R*′* = phenyl or hydrogen.

The three quantum chemically most stable conformers [2] of each of the LIGNOLs (three per stereoisomer for triphenyl) were chosen for this study. The four most relevant torsional angles in the LIGNOLs were properly analyzed during the simulations. Those are explained in detail in Figure 2, which also shows the numbering of atoms.

### 2.1. Solvation Effects in *Ab Initio* Calculations

It is well known that the surrounding medium might determine which conformation will be preferred. In order to corroborate this, the systems were studied in aqueous solution with the COSMO model at different levels of theory.

In Tables 1 and 2 the total electronic energies and the dipole moments, respectively, are expressed. The energies are given relative to the most stable conformer for each method used. The first two columns [2] show the total electronic energies or dipole moments in the gas phase. In the second column the level of theory used was B3LYP/TZVP. In the last two columns the values from the studies in the presence of water (*ɛ_r_* = 78.39) using the COSMO model are reported. The methods for the COSMO calculations in the third column is reoptimization at the restricted Hartree–Fock (RHF)/SV(P) level, and in the fourth column reoptimization with density functional theory (DFT) at the B3LYP/TZVP level of theory, *i.e*., the starting structures for the optimizations are the HF gas phase structures. The exact values of the torsional angles can be found in Table 3.

As can be seen in Table 1 the energies from the COSMO calculations follow the same order as the conformers in the gas phase for diphenyl and tetraphenyl 1,4-diol. For triphenyl and tetramethyl 1,4-diol the case is different.

The trend, however, is the same that could be observed in the gas phase study in a previous work [2]. For the energetically more favourable conformers, a *π − π* interaction was formed between the phenyl ring at C7 and one of the phenyl rings at C9*′*. As stated in [2] the change was initialized by a turn of the phenyl ring at C9*′* for **3PhS3** and **3PhS7**. In **3PhR3** the torsional angle *α* was turned backwards by *≈* 100*°*, but the phenyl ring at C9*′* was to begin with in a correct position to form the desired *π − π* interaction. What is notable is that this trend can be seen already at the HF level (column 3) in the COSMO calculations.

For the tetramethyl conformer **4Met6** this could not be noticed at the HF/SV(P) level even if the DFT optimization changed the structure a little, though, causing a big energy yield. The phenyl ring at C7 tilts almost 50*°*, then allowing a change in the aliphatic six-membered ring from an envelope like conformation to a boat conformation. This also forces one of the methyl groups closer to the phenyl ring at C7, exactly as it happened in the gas phase study [2].

When considering the dipole moments in Table 2, calculated with GAMESS at HF/6-31G* level, one can observe that the order between the conformers is preserved almost entirely for each method used. Generally the dipole moment always increases when a polar molecule is solvated. This is of course also true for the solvation models, as can be seen in Table 2.

When examining the values in Table 2 more carefully, one can see that the diphenyl 1,4-diols are the ones that follow the energy trend the closest. The only exception is *μ_DFT_* in gas phase for **2Ph9**. For triphenyl and tetraphenyl 1,4-diol a trend can be seen that conformers with higher dipole moment are more stable than the other, especially according to the DFT calculations. This also holds for tetramethyl 1,4-diol, for which conformer **4Met6** has by far the highest dipole moment and is most stable in DFT.

### 2.2. Molecular Dynamics

The initial values of each of the four torsional angles mentioned in the Figure 2 are shown in Table 3. In Table 3 also the torsional angles in the DFT optimized structures [2] are shown, but the HF structures are the ones used as starting structures in the MD simulations. The torsional angles that change a lot during the DFT optimizations are marked in bold.

**Figure 3:**
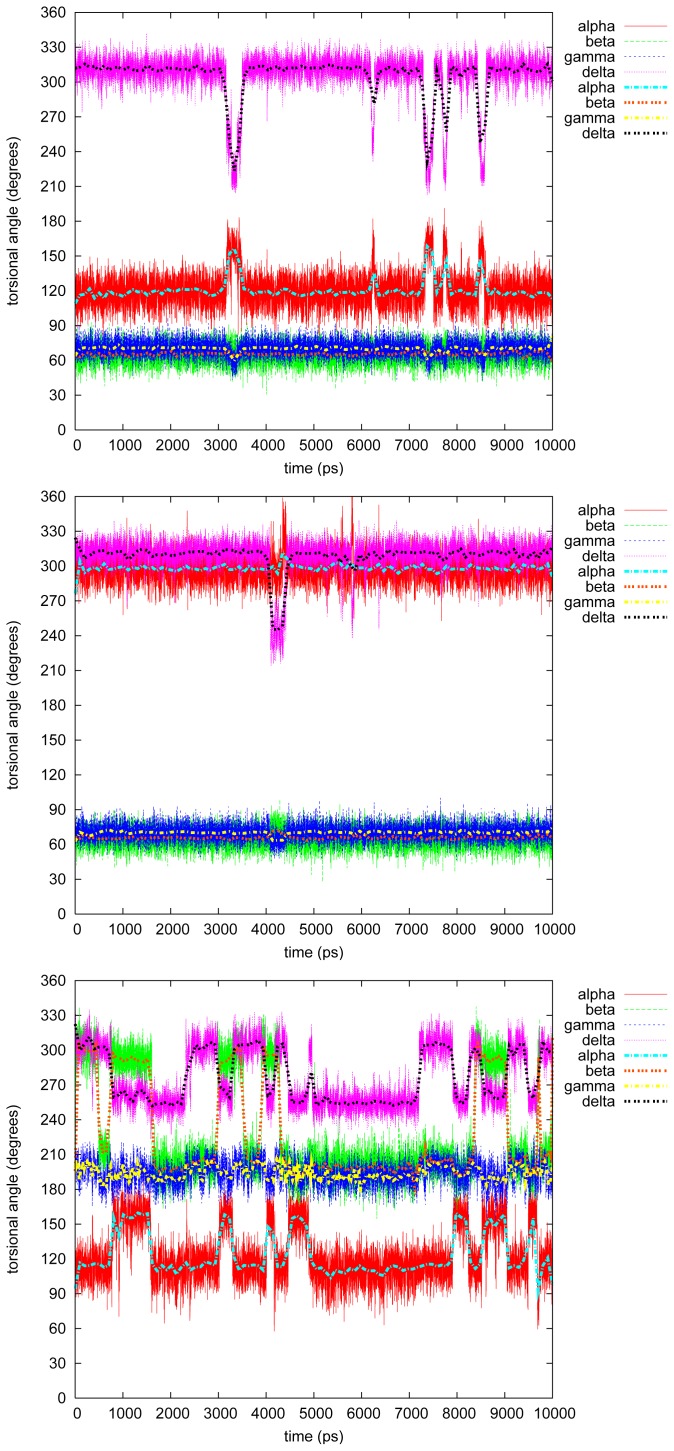
Torsional angles in diphenyl 1,4-diol.

**Figure 4:**
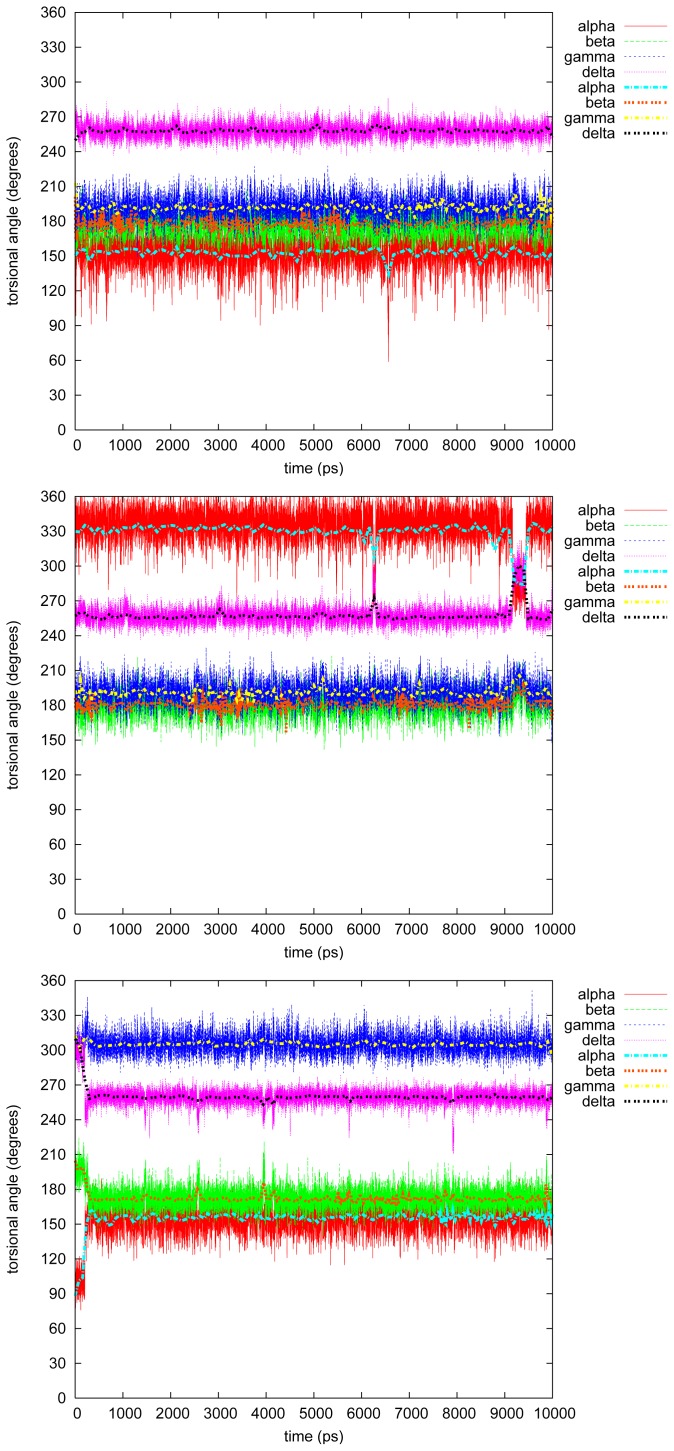
Torsional angles in triphenyl(R) 1,4-diol.

**Figure 5:**
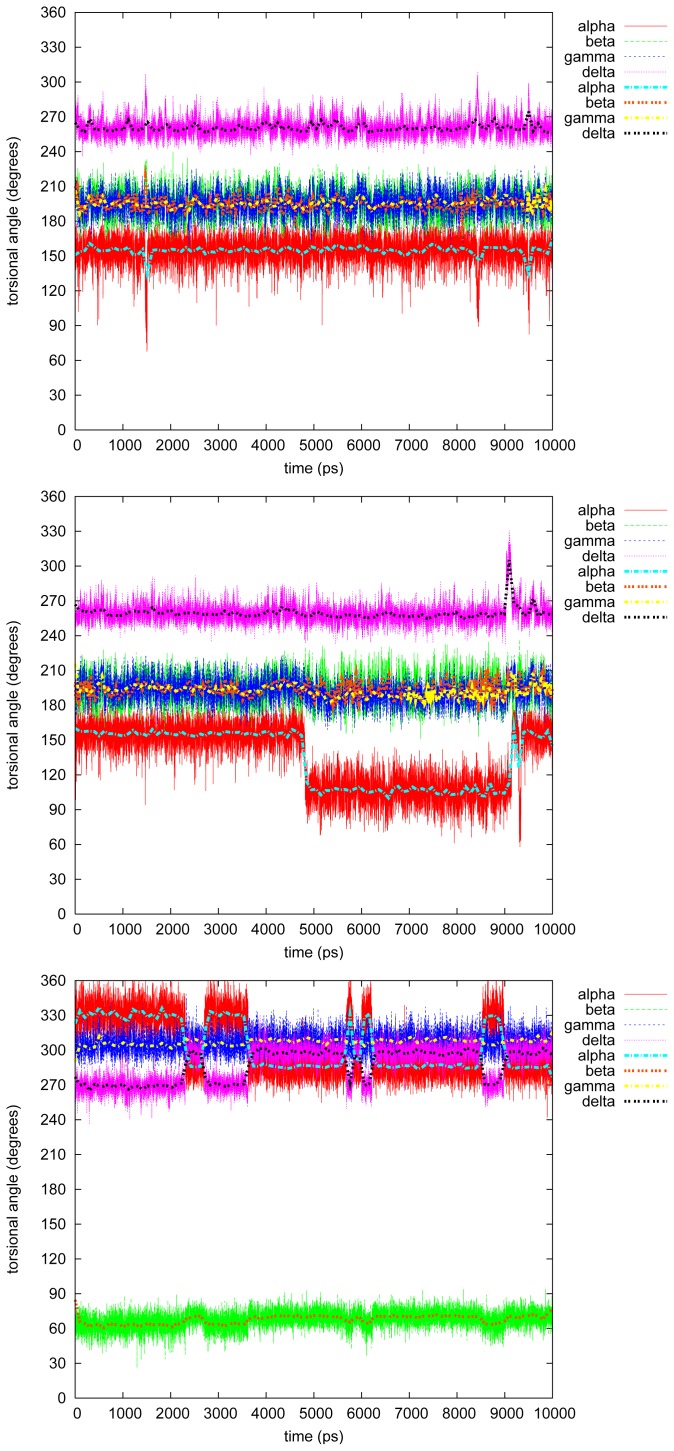
Torsional angles in triphenyl(S) 1,4-diol.

**Figure 6:**
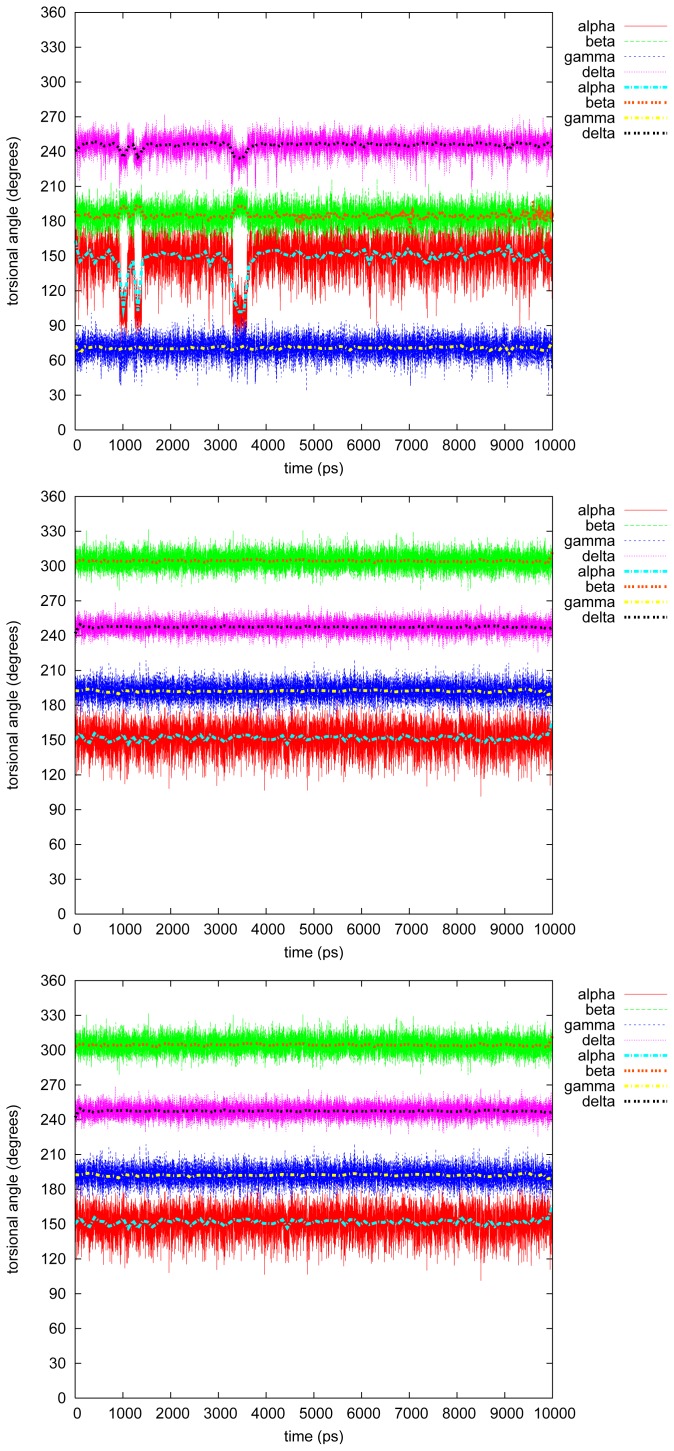
Torsional angles in tetramethyl 1,4-diol.

**Figure 7:**
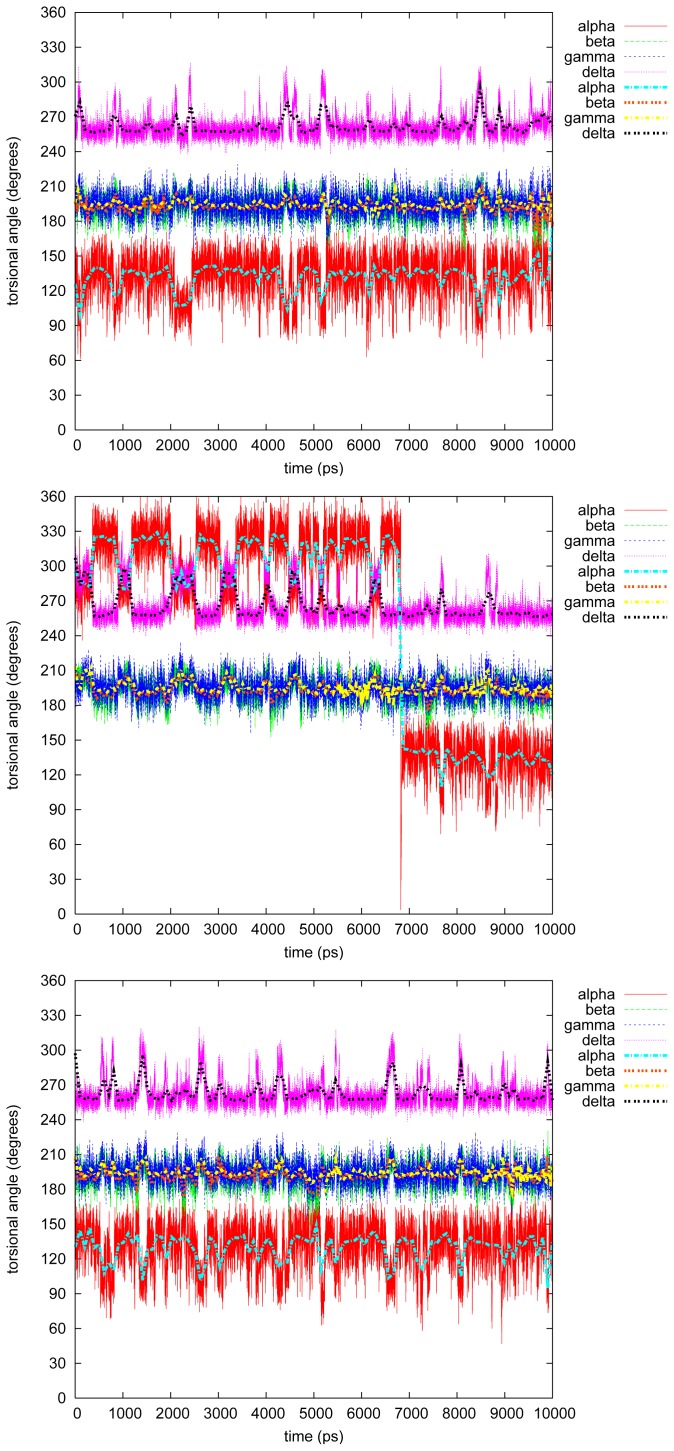
Torsional angles in tetraphenyl 1,4-diol.

Figures 3–7 show the changes in the four torsional angles during 10 ns simulations of the different conformations of the studied LIGNOLs. The Figures are plotted by *gnuplot, version* 4.0, and they include a smoothing (the thicker line) using the Bezier algorithm, *i.e*., an approximation of the data with a Bezier curve of degree *n* (where *n* equals the number of data points) that connects the endpoints. Each LIGNOL is analyzed separately.

#### 2.2.1. Diphenyl

The first two simulations for diphenyl 1,4-diol were almost *status quo*. The four torsional angles stayed close to their starting values; *α* = 120*°* or 300*°*, *β* = 60*°*, *γ* = 70*°* and *δ* = 315*°*, which can be seen in Table 3. In the first simulation a short conformational change occurs just after 3 ns into *α* = 150*°*, *δ* = 225*°* and *γ <* 60*°*, and this occurs a few times between 7 and 9 ns.

The third simulation of diphenyl 1,4-diol, however, is perhaps the most interesting case of all in this study. The torsional angle *γ* is as usual the most stable one in these LIGNOLs, staying at the starting value 195*°*. The highest populated conformation during this simulation is the one staying stable between 5 and 7 ns, with *α* = 120*°*, *β* = 200*°* and *δ* = 255*°*. This value of *δ* was found to make it possible for the aliphatic six-membered ring to be in boat conformation, by that raising the stability of the tri- and tetraphenylated 1,4-diols in [2]. The starting value of *β* = 200*°*, but this changes immediately to 300*°*. The angle *β* changes back at 0.5 ns not causing any immediate change, but perhaps initializing the change of *α →* 150*°* and *δ →* 255*°*. At 1.5 ns *α →* 120*°* and *β* back *→<* 200*°*. At 2.5 ns *δ →* 300*°* without any other consequences, but at 3 ns the conformation changes to *α* = 150*°*, *β* = 300*°* and *δ ≥* 255*°*. This does not stay for long until the conformation returns to *α →* 120*°* and *δ →* 300*°*, and soon afterwards, *β* back *→* 200*°*. This pattern continues until the population of the conformation raises at 5 ns. After 7 ns *δ* changes again *→* 300*°*, and at 8 ns the previous pattern from 3–5 ns starts again. If both *α* and *δ* change it seems to happen simultaneously, while *β* either initializes a conformational change or lags behind.

#### 2.2.2. Triphenyl(R)

The triphenyl 1,4-diol seems to be a much more hindered molecule than the diphenyl, as it also should be, especially the R stereoisomer which is discussed here. In all three simulations *δ* stays most of the time at *≈* 255*°*—the stabilizing value stated in [2]. The torsional angle *α* has the possibilities of 150*°* or 330*°*, with an exception in the second simulation, while *α →* 285*°* and *δ →* 300*°* at 9 ns, also raising *β* and *γ* with a few degrees, and the same quickly back and forth at 6.5 ns.

In the first two simulations *β* = 180*°* and *γ* = 195*°*, but in the third and last *β* is rather 165*°* and *γ* = 300*°*, even if the starting conformation here is *α* = 90*°*, *β* = 195*°* and *δ* = 300*°*. The torsional angle *γ* stays at 300*°* during the whole simulation.

#### 2.2.3. Triphenyl(S)

In the first two simulations of the triphenyl(S) stereoisomer, the conformations stay quite unchanged at *α* = 150*/*165*°*, *β* = *γ* = 195*°* and *δ* = 255*°*. Just before 5 ns in the second simulation *α →* 105*°*, but this does not seem to affect the rest of the torsional angles studied.

In the third and last simulation, triphenyl(S) 1,4 diol seems to flip between the conformers: *α* = 330*° − δ* = 270*°*, and *α* = 285*° − δ* = 300*°*. During the whole simulation *β* and *γ* stay at 60*°* and 300*°*, respectively, with just small fluctuations at the conformational change.

#### 2.2.4. Tetramethyl

A bit surprisingly tetramethyl 1,4-diol is the most hindered molecule of the LIGNOLs that were studied. Even the conformations are quite similar in the three simulations: *α* = 150*°*, *β* = 180*/*300*°*, *γ* = 75*/*195*°* and *δ ≥* 240*°*. In the first simulation, though, *α* changes three times *→* 105*°* (at 1 and 3.5 ns), but this has very little effect on the rest of the studied angles.

#### 2.2.5. Tetraphenyl

The originally planned end product in the synthesis route of the LIGNOLs, *i.e*., tetraphenyl 1,4-diol, is a more interesting case from the torsional point of view than the last one, even though, *β* = *γ* = 195*°* through all of the simulations. However, *α* and *δ* fluctuate most of the time between the conformers *α* = 135*/*330*°–δ* = 255*°*, and *α* = 105*/*285*°–δ* = 285*/*300*°*. In the second simulation just before 7 ns, *α* flips drastically*→* 135*°*, though not causing anything at all on the rest of the studied torsional angles.

#### 2.2.6. Discussion on the Simulations

For diphenyl 1,4-diol, the highest populated conformations seemed to be **2Ph1** and **2Ph2**, as can be seen in Table 2 in [8] and in Table 3 in this article. Another quite highly populated conformation in the simulations in TIP4P water is **2Ph9**, which is the third one picked out as starting geometry for the simulations, but with the exception that the torsional angle *δ* is rather the favourable 255*°* than the starting value *≈* 315*°*. However, this change of *δ* seems to imply that *α* takes the uncommon value of 150*°*, also causing or implying *β* to be 300*°*.

In the case of triphenyl 1,4-diol, especially the R stereoisomer, it is more comprehensible that the value 150*/*330*°* occurs for *α* as there is an additional phenyl ring substituted on C9. The highest populated conformers could be said to be *≈* the DFT optimized **3PhR3**, and **3PhS3** and **3PhS7**, respectively, even though the values for the S stereoisomers vary a bit from the optimized ones. The small variation in *β* and *γ* from the optimized ones might, though, be explained by the sensitivity of them, when forming hydrogen bonds between the hydroxyl groups, O9–H and O9*′*–H, and the TIP4P water. The torsional angles in the highest populated conformer in the last triphenyl(S) simulation is actually very close to **3PhS10**.

In tetramethyl 1,4-diol it is simply the three most stable conformers in gas phase that dominate, *i.e.*, **4Met2**, **4Met3** and **4Met6**, the last two of which are actually the same conformer.

The last molecule studied, tetraphenyl 1,4-diol, is trickier to summarize in the system of the conformers optimized in gas phase. The most frequent value of *δ* in the simulations, 255*°*, only occurs for the conformers **4Ph5** and **4Ph6**, but for those the other torsional angles vary a lot. The only conformer of the simulations, fitting well with the gas phase values, is **4Ph4** and **4Ph8**, actually the same, which occurs in the second simulation. The big fluctuations compared to the other LIGNOLs might, though, be a sign of various populations of the molecule.

Opposite to the conclusion for HMR [8], the quantum chemical calculations (in gas phase), thus, seemed to predict reliable trends for how these molecules act in solvents. Perhaps this was more reliable as the molecules were bigger than HMR.

#### 2.2.7. Hydration Effects

In the LIGNOLs there are four hydrogen bonding acceptor oxygen atoms in methoxy groups. However, the most interesting oxygens from the reaction point of view are those in the metal-complexing hydroxyl groups, O9–H and O9*′*–H. In those groups there are also two hydrogen bonding donors.

In order to understand the hydration effect more properly, the g_hbond analysing program implemented in GROMACS was used to study the number of hydrogen bonds for the oxygen atoms O9 and O9*′*, and totally for each LIGNOL conformer, as well as the average lifetime of the uninterrupted hydrogen bonds, which are all shown in Table 4.

In a previous study [8] the average number of hydrogen bonds per time frame for the oxygen atoms in the methoxy groups was *≈* 0.24, which means that the four methoxy oxygens in the LIGNOLs contribute with one hydrogen bond altogether. The rest consists mainly of contributions from the reactive centre, *i.e*., the hydroxyl groups, O9–H and O9*′*–H.

The first three columns in Table 4 show that O9 seems to be a bit more likely to form hydrogen bonds to the solvent. This conclusion might, though, be erroneous due to the fact that the hydroxyl groups O9–H and O9*′*–H form an internal hydrogen bond, which takes away one connection to the solvent, for those conformers that have the OH groups pointing in the same direction. However, it is notable that tetramethyl 1,4-diol is more likely to form hydrogen bonds to TIP4P and tetraphenyl is less likely so, mainly due to the small tendency of O9 to form hydrogen bonds to TIP4P.

Considering the lifetimes, in [8] it was stated that the average lifetime of the uninterrupted hydrogen bonds was calculated to be 1.2 ps. The last column shows that this also holds quite well for the LIGNOLs. A few are just below 1 ps. When looking at columns 4 and 5, more interesting values can be noticed, especially for O9*′* (col. 5). A correlation can, however, be seen to the number of hydrogen bonds as the lifetimes are longer for tetramethyl 1,4-diol and shorter for tetraphenyl. A shorter lifetime for a large average number of hydrogen bonds may imply that they are quite weak, meaning that the hydrogen bonds from O9*′* in **2Ph1** and **2Ph2** might be strong. This again could be very important for the application of these LIGNOLs as metal-binding agents, as the bonding to a metal-atom catalyst would act as the hydrogen bonding to TIP4P water. Diphenyl 1,4-diol is the only LIGNOL in this study with phenyls at C9*′* and not at C9, so the reason for this phenomenon is probably the electronic effects of the phenyl rings at C9*′*.

The hydrogen bond lengths were also analyzed by using g_hbond, and the mean value of the hydrogen bond lengths is approximately 0.28 nm, exactly as in [8].

## 3. Computational Methods

The three quantum chemically most stable conformers [2] of each of the LIGNOLs were placed in water-like continuum solvent (*ɛ_r_* = 78.39) using COSMO [9,10] in the TURBOMOLE program package [14,15] version 6.1. The structures in continuum solvent were reoptimized at the RHF level [16,17] with the basis set SV(P) [18], to be comparable with the RHF calculations in [2], where the basis set 6-31G* [19–21] was used. After that the structures were reoptimized using DFT [22] with the B3LYP hybrid exchange-correlation functional [23–25] in combination with the MARI-J approximation [26–28] and the TZVP basis set [29] for all atoms, as implemented in the TURBOMOLE program package.

The MD simulations were performed using GROMACS version 4.5.3 software [30–34]. Water was described using the TIP4P model [35], and the LIGNOLs were modeled with the OPLS-AA force field [36] implemented in GROMACS. The topologies of the LIGNOLs were constructed manually, and they comprised 415 (2Ph), 474 (3Ph), 533 (4Ph) and 369 (4Met) internal coordinates, respectively. In order to get reasonable atomic charges to help for choosing suitable atom types with the hand-tuned charges available in the force field, electrostatic potential fit (ESP) charges were studied with GAMESS at HF/6-31G* level. For O9 and O9*′* (shown in Figure 2) the OPLS atom type opls_154 with the atomic charge *–*0.683 was found to be the most suitable one, and for the other four oxygens (O3, O4, O4*′* and O5*′*) the atom type opls_179 with the atomic charge −0.285 was chosen. An important detail to consider is also that the sum of the atomic charges in a charge group should be an integer or equal to zero.

The initial (quantum chemically optimized) conformations of the LIGNOLs were taken from our previous work [2]. Each conformation was placed at the center of a cubic box with the dimension between 5.2 and 5.6 nm (volume = 144–174 nm^3^) and solvated by 4802–5795 water molecules. Each system was first energy minimized < 2000 kJ mol*^−^*^1^ nm*^−^*^1^ using steepest descent for 3–121 steps. Then the system was equilibrated at 398 K for 50 ps, and finally the production simulation was run for 10 ns with the temperature maintained at 298 K using the Berendsen thermostat [37]. The pressure was maintained at 1 atm using the Berendsen barostat [37]. A 1 fs time step was used in all simulations. A cutoff of 0.9 nm was applied to short-range nonbonded interactions, and for long-range electrostatic interactions the particle mesh Ewald (PME) method [38,39] was used with grid spacing of 0.12 nm and fourth-order interpolation. In all simulations system snapshots were collected every 500 steps, *i.e*., 0.5 ps, for subsequent analysis. In this time only electronic excitations and bonding vibrations will occur, but those can be ignored when studying the conformational preferences of the system.

## 4. Conclusions

- In MD simulations on the LIGNOLs, the conformations preferred were the energetically most favourable ones according to quantum chemical DFT calculations in gas phase, almost irrespective of the dipole moment.
- The four most relevant torsional angles *α − δ*, defined in Figure 2, varied during dynamics in accordance with their symmetry.
- The torsional angle *δ* was generally more preferred at the stabilizing value 255*°* than what was seen in the gas phase optimizations in [2].
- No strong correlation patterns were found, but in the last simulation of **2Ph9**, *α* and *δ* changed simultaneously, while *β* either initialized a conformational change or lagged behind.
- In the hydration studies **2Ph1** and **2Ph2** were found to have strong hydrogen bonds from O9*′*, which could be very important for the application of these LIGNOLs as metal-binding agents.
- The hydration studies of the MD simulations show that several of these LIGNOLs, produced from a renewable source, have a great potential of acting as chiral catalysts.

## Figures and Tables

**Table 1:** Relative energies in kJ/mol including solvation effects.

Conformation	*E_HF_* [2]	*E_DFT_* [2]	*E_COS,HF_*	*E_COS,DFT_*
2Ph1	6.1	7.3	5.7	9.4
2Ph2	5.2	6.7	4.2	8.1
2Ph9	0	0	0	0
3PhR3	21.2	0	0	0
3PhR4	20.5	24.5	28.6	22.5
3PhR5	27.8	36.1	46.2	44.5
3PhS3	35.5	11.6	22.0	12.7
3PhS7	19.6	11.7	25.2	12.6
3PhS10	0	12.1	15.9	17.3
4Met2	8.9	4.5	4.7	7.6
4Met3	0	4.2	0	9.2
4Met6	39.9	0	27.9	0
4Ph3	0	0	0	0
4Ph4	2.0	2.0	1.7	3.9
4Ph8	0.9	1.5	0.9	2.9

**Table 2:** Dipole moments in Debye including solvation effects.

Conformation	*μ_HF_*	*μ_DFT_*	*μ_COS,HF_*	*μ_COS,DFT_*
2Ph1	2.19	2.11	3.88	4.07
2Ph2	1.93	1.56	3.36	3.38
2Ph9	1.79	2.48	1.87	2.54
3PhR3	6.54	4.40	6.08	6.44
3PhR4	3.46	3.92	4.22	5.22
3PhR5	2.80	2.09	3.85	3.77
3PhS3	6.61	6.71	9.29	9.51
3PhS7	6.64	6.67	8.90	9.50
3PhS10	2.22	2.07	3.57	3.72
4Met2	2.03	2.27	2.50	3.42
4Met3	1.90	2.09	3.04	3.55
4Met6	5.33	3.34	7.48	5.48
4Ph3	6.45	6.50	8.40	9.35
4Ph4	3.91	3.85	5.73	6.24
4Ph8	3.48	3.13	4.24	4.12

**Table 3:** The initial (HF) and the DFT optimized torsional angles.

Conformation	Method	*α*	*β*	*γ*	*δ*
2Ph1	HF	115.9*°*	49.6*°*	78.3*°*	314.5*°*
	DFT	115.6*°*	49.5*°*	78.3*°*	313.5*°*
2Ph2	HF	294.9*°*	48.8*°*	78.2*°*	315.0*°*
	DFT	294.2*°*	49.7*°*	78.1*°*	314.4*°*
2Ph9	HF	117.1*°*	198.3*°*	194.8*°*	311.8*°*
	DFT	117.3*°*	196.7*°*	195.3*°*	310.8*°*
3PhR3	HF	90.0*°*	190.4*°*	198.8*°*	285.0*°*
	DFT	**348.8***°*	**160.3***°*	180.2*°*	**256.8***°*
3PhR4	HF	280.5*°*	190.8*°*	200.3*°*	285.8*°*
	DFT	270.9*°*	196.3*°*	198.3*°*	280.9*°*
3PhR5	HF	103.5*°*	206.4*°*	301.1*°*	302.4*°*
	DFT	101.0*°*	209.8*°*	303.5*°*	297.9*°*
3PhS3	HF	116.7*°*	224.4*°*	143.9*°*	305.1*°*
	DFT	**170.8***°*	216.4*°*	**185.3***°*	**267.6***°*
3PhS7	HF	128.6*°*	208.1*°*	198.8*°*	299.6*°*
	DFT	**171.4***°*	215.6*°*	186.2*°*	**268.3***°*
3PhS10	HF	344.0*°*	64.8*°*	293.3*°*	260.9*°*
	DFT	344.4*°*	64.5*°*	292.2*°*	260.5*°*
4Met2	HF	166.0*°*	177.9*°*	66.6*°*	247.1*°*
	DFT	167.8*°*	180.6*°*	65.1*°*	245.7*°*
4Met3	HF	163.8*°*	305.8*°*	187.7*°*	248.2*°*
	DFT	165.6*°*	307.8*°*	187.3*°*	247.0*°*
4Met6	HF	117.6*°*	326.2*°*	196.8*°*	294.9*°*
	DFT	**166.3***°*	307.1*°*	187.6*°*	**247.0***°*
4Ph3	HF	87.7*°*	204.3*°*	193.2*°*	289.0*°*
	DFT	84.0*°*	208.6*°*	190.2*°*	283.3*°*
4Ph4	HF	280.2*°*	203.3*°*	196.5*°*	289.2*°*
	DFT	280.4*°*	205.4*°*	196.3*°*	286.0*°*
4Ph8	HF	275.3*°*	203.3*°*	195.1*°*	290.0*°*
	DFT	273.8*°*	205.1*°*	194.8*°*	287.1*°*

**Table 4:** The average number of hydrogen bonds per time frame and the average lifetime (ps) of the uninterrupted hydrogen bonds between the LIGNOLs and the TIP4P solvent.

Conformation	*num_O_*_9_	*num_O_*_9_*′*	*num_Tot_*	*life_O_*_9_	*life_O_*_9_*′*	*life_Tot_*
2Ph1	1.24	0.98	6.23	3.35	**6.18**	1.16
2Ph2	1.23	0.98	6.26	3.26	**5.82**	1.15
2Ph9	1.41	0.80	6.08	2.29	2.24	1.06
3PhR3	1.16	0.71	5.54	2.03	1.67	0.97
3PhR4	1.11	0.78	5.53	1.97	1.72	0.98
3PhR5	1.27	1.15	6.08	2.88	3.15	1.11
3PhS3	1.25	0.65	5.58	2.08	1.58	0.99
3PhS7	1.12	0.80	5.60	1.96	1.78	0.98
3PhS10	1.10	1.14	6.51	3.53	3.35	1.19
4Met2	1.20	**1.33**	6.35	3.95	3.63	1.18
4Met3	1.37	**1.24**	**7.05**	3.57	3.83	1.27
4Met6	1.37	**1.24**	**7.04**	3.56	3.86	1.28
4Ph3	**0.62**	1.05	5.31	1.57	2.07	0.95
4Ph4	**0.68**	0.95	5.23	1.82	2.00	0.96
4Ph8	**0.79**	0.85	5.26	1.88	1.77	0.94
